# Automatic and Objective Assessment of Alternating Tapping Performance in Parkinson's Disease

**DOI:** 10.3390/s131216965

**Published:** 2013-12-09

**Authors:** Mevludin Memedi, Taha Khan, Peter Grenholm, Dag Nyholm, Jerker Westin

**Affiliations:** 1 School of Technology and Business Studies, Computer Engineering, Dalarna University, Falun SE-791 88, Sweden; E-Mails: tkh@du.se (T.K.); jwe@du.se (J.W.); 2 School of Science and Technology, Örebro University, Örebro SE-701 82, Sweden; 3 School of Innovation, Design and Technology, Mälardalen University, Västerås SE-721 23, Sweden; 4 Department of Neuroscience, Neurology, Uppsala University, Uppsala SE-751 85, Sweden; E-Mails: peter.grenholm@akademiska.se (P.G.); dag.nyholm@neuro.uu.se (D.N.)

**Keywords:** alternating tapping, touch-pad, handheld computer, telemedicine, Parkinson's disease, remote monitoring, automatic assessment, objective assessment, visual assessment

## Abstract

This paper presents the development and evaluation of a method for enabling quantitative and automatic scoring of alternating tapping performance of patients with Parkinson's disease (PD). Ten healthy elderly subjects and 95 patients in different clinical stages of PD have utilized a touch-pad handheld computer to perform alternate tapping tests in their home environments. First, a neurologist used a web-based system to visually assess impairments in four tapping dimensions (‘speed’, ‘accuracy’, ‘fatigue’ and ‘arrhythmia’) and a global tapping severity (GTS). Second, tapping signals were processed with time series analysis and statistical methods to derive 24 quantitative parameters. Third, principal component analysis was used to reduce the dimensions of these parameters and to obtain scores for the four dimensions. Finally, a logistic regression classifier was trained using a 10-fold stratified cross-validation to map the reduced parameters to the corresponding visually assessed GTS scores. Results showed that the computed scores correlated well to visually assessed scores and were significantly different across Unified Parkinson's Disease Rating Scale scores of upper limb motor performance. In addition, they had good internal consistency, had good ability to discriminate between healthy elderly and patients in different disease stages, had good sensitivity to treatment interventions and could reflect the natural disease progression over time. In conclusion, the automatic method can be useful to objectively assess the tapping performance of PD patients and can be included in telemedicine tools for remote monitoring of tapping.

## 1. Introduction

The ability to perform functional upper limb motor tasks is essential for most of activities of daily living. Patients diagnosed with Parkinson's disease (PD) often have difficulties with timing control and coordination of upper limb movements [1,2]. In this neurodegenerative condition, patients also experience some of the four cardinal symptoms of PD including rigidity, bradykinesia (slowness of movements), postural instability and tremor.

Measuring symptoms and treatment-related complications in advanced PD is challenging. In clinical settings today, quantification of PD symptoms is usually done by employing rating scales, like the Unified Parkinson's Disease Rating Scale (UPDRS), which is mainly based on observations and judgments by clinicians. During evaluation of symptoms, both the clinician- and patient-derived outcome measures offer complementary information. The gold standard approach to evaluate the severity of upper limb motor symptoms is to use the UPDRS-part III (motor examination), more specifically items #23 (Finger Tapping), #24 (Hand Movements) and #25 (Rapid Alternating Movements of Hands) [3]. However, the use of these clinical scales is not suitable for long-term and repeated follow-up of symptoms since they are relatively time consuming [4], may need to be filled out at a clinic visit which in turn may influence patient outcomes [5], require considerable clinical experience [6] and some of their items have poor inter-rater reliability [7,8].

Therefore, there is a need for objective and observer-independent measurements which may provide better resolution than clinical scales for more accurately capturing symptom severities and fluctuations. Quantitative measurement of upper limb motor performance of PD patients during finger tapping tests has been previously analyzed by the use of musical instrument keyboard [6], personal computer keyboard [9], magnetic sensors [10,11], optoelectronic camera [12], infra-red emitting diodes [13], computerized assessment battery [14] and accelerometry [15]. The results from these studies showed that objective measures of finger tapping performance correlated well with clinical ratings scores indicating that these technologies contained important elements of the information of the well-established scales. As in the case of outcome measures derived by clinical rating scales, objective measures derived by computer assessment tools should be scientifically sound in terms of validity, reliability and sensitivity to change [16–18].

This paper presents the development and evaluation of a method for enabling quantitative and automatic scoring of alternating tapping performance (ATP) of PD patients, using a touch-pad handheld computer designed for telemedicine. The paper reports on different metrics to evaluate the quality of assessments of the method including correlations to visually assessed scores of ATP and UPDRS motor ratings, reliability, and sensitivity to treatment interventions and natural PD progression over time. In addition, the ability of the method to discriminate between healthy elderly subjects and patients in different disease stages is reported.

## 2. Methods

### 2.1. Subjects

The results presented in this paper are based on data from two clinical studies, both of which were approved by the relevant agencies and written informed consent was given. In total, 95 patients in different clinical stages of PD and 10 healthy elderly (HE) subjects were assessed (Table 1).

Sixty-five patients diagnosed with advanced PD were recruited in an open longitudinal 36-months study (Duodopa in Advanced Parkinson's: Health Outcomes & Net Economic Impact, EudraCT No. 2005-002654-21) at nine clinics around Sweden [19]. On inclusion, 35 of them were treated with levodopa-carbidopa gel intestinal infusion (LCIG) and 30 patients were candidates for switching from conventional oral PD treatment to LCIG (hereafter denoted as LCIG-naïve group). In the second study, 30 patients with a clinical diagnosis of idiopathic PD in Milan, Italy, participated [20]. The Italian study included two patient groups: intermediate stage patients experiencing on-off fluctuations (F group) and clinically stable patients (S group).

### 2.2. Telemetry Assessments

**Figure 1.**
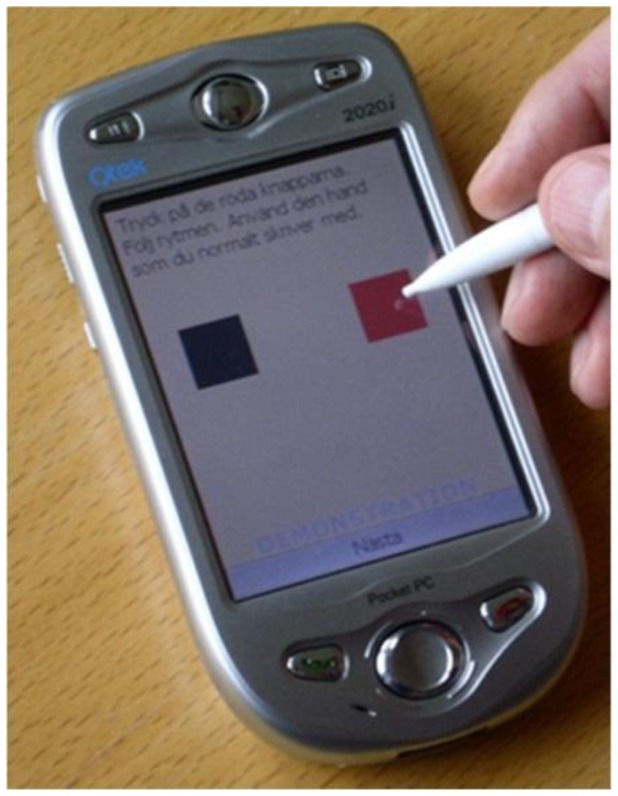
Illustration of the alternating tapping test using the telemetry device.

Both patients and HE subjects performed repeated and time-stamped assessments in their home environments using a telemetry test battery implemented on a touch-pad handheld computer [21]. On each test occasion, they were asked to perform objective tests of their upper limb motor performance including 20 seconds-long uncued alternate tapping of two square areas (“fields”) (Figure 1). The fields had a side of approximately 15 mm and were located 27 mm apart. The fields were shown on the screen and subjects were instructed to use an ergonomic pen stylus to tap as fast and accurate as possible, using first right hand and then left hand. The subjects were instructed to place the handheld computer on a table and to be seated in a chair. Raw telemetry data, consisting of tap position (x-y pixel coordinates) and timestamps (in milliseconds;, were collected and wirelessly transmitted in an XML format to a central server for storage and offline processing. The test battery was implemented on a Qtek 2020i Pocket PC device having a 3.5” touch screen and a 240 × 320 pixel resolution.

Assessments with the test battery were performed four times per day during week-long test periods. In the Swedish study, the test battery was used quarterly for the first year and biannually for the second and third years. The LCIG-naïve patients used the test battery at baseline (in which they were on oral treatment), month 0 (first visit; at least 3 months after percutaneous endoscopic gastronomy surgery), and at follow-up test periods. In 23 LCIG-naïve patients, assessments with the test battery were available during oral treatment and at least one test period after having started infusion treatment. Hence, n = 23 in the LCIG-naïve group.

In the Italian study, patients used the test battery for two test periods with a washout week in between. The HE subjects used the test battery for one test period. The total number of observations with the test battery were as follows: Swedish group (n = 10,079), Italian F group (n = 822), Italian S group (n = 811), and HE (n = 299).

The development and evaluation of the method was mainly done using the Swedish dataset. To avoid onset and offset effects, data points collected during the first and last two seconds of the test time were discarded. Hence, the time series of interest were in the range between 2 s and 18 s.

### 2.3. Visual Assessment of ATP

A web-based system was developed to visualize the performance of patients during tapping tests and to allow users (PD specialists) to rate different tapping impairments [22]. The system was designed as a three-tier web application using JavaServer Pages and MySQL Server as a back-end database.

**Figure 2.**
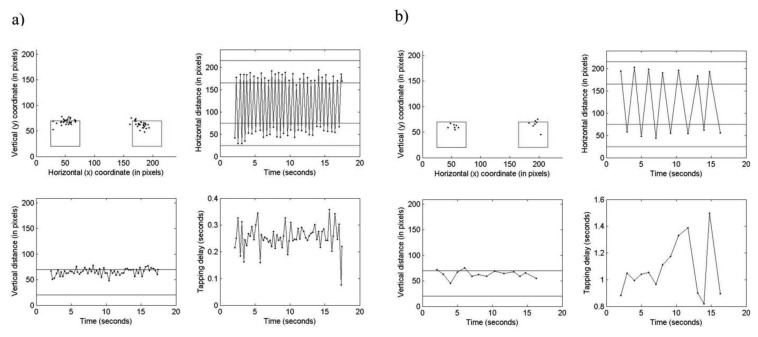
Two illustrative examples of visualized ATP in the web-based system. (**a**) A test occasion with V-SPEED, V-ARRHTYHMIA, V-FATIGUE and V-GTS rated 0 (normal) and V-ACCURACY 1 (mild). (**b**) A test occasion with V-SPEED rated 4 (extremely severe), V-ACCURACY 1 (mild), V-FATIGUE 3 (severe), V-ARRHYTHMIA 0 (normal) and V-GTS 4 (extremely severe). The left field is represented with blue color. The right field is represented with red color.

The system retrieved time series of raw data from the database tables and visually depicted them into different types of graphs. Information presented included: (i) distribution of taps over the two fields; (ii) horizontal tap distance *vs.* time; (iii) vertical tap distance *vs.* time; and (iv) tapping reaction time over the test length (Figure 2). A neurologist was instructed first to visually interpret the tapping variation, patterns and trends within the graphs, and then to assess the observed impairments on 0 (normal) to 4 (extremely severe) categorical scales. First ratings of four tapping dimensions, including ‘speed’, ‘accuracy’, ‘fatigue’ and ‘arrhythmia’, were done followed by rating of the ‘global tapping severity’ (GTS). The dimensions were considered specific for the type of movement disorder found in PD patients, as defined by the items 23–25 of the UPDRS-III (finger tapping, hand movements, rapid alternating of movements, respectively) [3]. The ‘speed’ dimension measured the ability to tap rapidly during the test. Cases that had a large number of taps were rated as normal (0) whereas those with very low number of taps were rated as extremely severe (4). The subject's ability to correctly tap the fields on the screen was measured by the ‘accuracy’ dimension. Normal cases were considered those which had majority of taps within the fields and not widely spread whereas extremely severe cases were considered those which had majority of taps outside the fields followed by a larger spread. The amount of tapping irregularity and the progressive reduction of movements across the tap test were measured by ‘arrhythmia’ and ‘fatigue’ dimensions, respectively. The ‘fatigue’ dimension was visualized as increment in the tapping delay graph where if the tapping was regular and the curve was flat the case was rated normal whereas if there was a continuous slowing with longer delays between each tap the case was rated extremely severe. For the ‘arrhythmia dimension, cases that exhibited no interruptions or arrests in the movements were rated as normal whereas cases that exhibited frequent interruptions and multiple taps in the same field were rated as extremely severe. The GTS was assumed to be a composite score of the four dimensions providing a holistic representation of the patient's ATP. The visually assessed scores are hereafter denoted as V-SPEED, V-ACCURACY, V-FATIGUE, V-ARRHYTHMIA and V-GTS. The web-based system visualized at least 20 test occasions per each GTS level to the neurologist.

### 2.4. Computerized Assessment of ATP

In total, 24 quantitative parameters (Table 2) were extracted from time series data to represent patients' symptom severities during tapping tests, using time series analysis and statistical methods. The data was summarized into scores for the four tapping dimensions and the GTS.

#### 2.4.1. Calculation of Automated Speed Score (A-SPEED)

To quantify the ‘speed’ performance during tapping tests, the following parameters were calculated and used in the subsequent analysis. The total number of taps (TNT) was calculated as the total sum of taps in a test occasion for the mid 16 s. The mean tapping speed (MTS) was defined as the mean rate of change of tap distance with time, using the following Equation:

(1) $$\text{MTS}=\sum_{i=1}^{n}\frac{\sqrt{{\left(x_{i+1}-x_{i}\right)}^{2}+{\left(y_{i+1}-y_{i}\right)}^{2}}}{t_{i+1}-t_{i}}$$

where *n* is the total number of taps in a test occasion, *x* is the horizontal coordinate of pixels on the touch screen, *y* is the vertical coordinate and *t* is time in milliseconds. In order to catch side variability of ‘speed’ while tapping the two fields, mean and coefficient of variation (defined as the ratio between standard deviation and mean, CV) of tapping speed for both sides were calculated. The side, left to right (LR) or right to left (RL), was depicted using the following Equation:

(2) $$\text{Side}=\{\begin{array}{cc} LR, & x_{i}<x_{i+1}\\ RL, & x_{i}>x_{i+1} \end{array}$$

Hence depending on the side, the following parameters were calculated: mean tapping speed from left to right (MTSLR), CV of tapping speed from left to right (CVTSLR), mean tapping speed from right to left (MTSRL) and CV of tapping speed from right to left (CVTSRL). Principal Component Analysis (PCA) using correlation matrix method was applied to these parameters to reduce their dimensions and obtain a single parameter. The purpose of PCA is to take *n* variables *X_1_*, *X_2_*, …, *X_n_*, find combinations of these and transform them into a new set of non-correlated variables *Z_1_*, *Z_2_*, …, *Z_n_*, called principal components (PCs) [23]. These components are linear combinations of the original variables and are derived in the decreasing order of eigenvalues so that the first PC accounts for the largest possible variance in the data. When dealing with multivariate applications, PCA represents the original data in the first two or three components thus enabling better understanding of the data as well as operating with a small number of variables in the subsequent analyses. The first PC of the parameters accounted for 69% of the total variance in the original data and was used to represent the A-SPEED.

#### 2.4.2. Calculation of Automated Accuracy Score (A-ACCURACY)

As stated above, this dimension reflects the subject's ability to correctly tap the fields on the screen and mainly focuses on coordination deficits. To quantify the ‘accuracy’ during tapping, the following four parameters were calculated. To measure the overall precision while tapping the two fields over the test trial, the mean distance from the centers of the fields (MDCF) was calculated. For the taps that were tapped within the area of the fields, the distance was preset to zero. The second parameter measures the regularity of precision over the test trial and is defined as the CV of distances from the center fields (CVDCF). The higher the CVDCF, the higher irregularity of tapping precision is. In order to quantify the overall distribution of the taps (ODT) over the two fields, initially the variation (ratio between summed distance and total number of taps) for each field was calculated followed by a calculation of mean variation of the two fields. Finally, the overall tapping precision (OTP) was defined as the mean distance from center fields irrespective of whether the taps were inside or outside the field areas, corrected for total number of taps. After applying PCA to these four parameters, the first PC accounted for 65% of the variance in the data and was used to represent the A-ACCURACY.

#### 2.4.3. Calculation of Automated Fatigue Score (A-FATIGUE)

The ‘fatigue’ during tapping is usually characterized by continued demotion of tapping performance relative to the passage of the test trial. The following parameters were defined to quantify the ‘fatigue’ dimension. The first parameter is the mean tapping speed per cycle (MTSPC) and was calculated as follows Equation:

(3) $$\text{MTSPC}=\sum_{i=1}^{n}\frac{D_{i+1}+D_{i}}{\Delta t_{i+1}+\Delta t_{i}}$$

where *n* is the total number of taps, *D* is the distance between adjacent taps defined as $\sqrt{{\left(x_{i+1}-x_{i}\right)}^{2}+{\left(y_{i+1}-y_{i}\right)}^{2}}$ and *Δt* is the change in time defined as *t_i_*_+_*_1_*– *t_i_*. The tapping cycle is defined as the movement from one field to the other and backwards. In order to capture demotion in tapping performance over the test trial, the time series signal was initially divided into two parts and then analyzed as follows. The first part consisted of data points sampled during the test time 2–10 s and the second part during the test time 10–18 s. The absolute mean differences between the first and second part of the time series were calculated for *Δt* and *MTSPC* resulting in two more parameters denoted as DDT12 and DMTSPC12, respectively.

To quantitatively characterize any change in sequential irregularity (or aperiodicity) of time series between the first and second part of the signal, Approximate Entropy (ApEn) statistical measure was applied. ApEn measures the similarity between a chosen window of time series of a given duration and the next set of windows of the same duration. A time series containing a single frequency component has a relatively small ApEn value whereas more complex time series containing multiple frequency components have high ApEn values, as a result of high level of irregularity. A detailed description of ApEn method can be found elsewhere [24]. Given a time series with *N* data points, ApEn requires determination of two user-specified parameters: a length of the window *m* and a measure of similarity *r*, each of which must remain fixed in all calculations. In this work, *m* was set to 2 and *r* to 0.2 (20% of the time series' standard deviation), as suggested by Pincus in [24]. In order to measure change in timing irregularity over the test trial, initially ApEn was applied separately on *Δt* of the two parts of the time series signal, that is first (2–10 s) and second (10–18 s), and then their mean absolute difference was calculated and used as a new parameter denoted as DAEDT12.

The other two parameters used for measuring tapping ‘fatigue’ were based on Dynamic Time Warping (DTW) method. The DTW is an algorithm used for comparing time series of different lengths and speeds by first locally stretching or compressing them and then by “warping” their time axes so that a relationship between the data points in the time series is maintained. Given two discrete time series, an input *A* = (*a_1_*, *a_2_*, …, *a_N_*), where *i* = *1*…*N*; and a reference *B* = (*b_1_*, *b_2_*, …, *b_N_*), where *j* = *1*…*M*, the DTW compares them as follows. The first step is to calculate absolute local dissimilarities between paired *i*th data points of *A* and *j*th data points of *B*, leading to a construction of a cross-distance matrix (*d*). The matrix *d* has small values if the data points are similar and large values if they are different. Next, an alignment path (or warping path) is created using a warping function *w*(*k*) = (*w_a_*(*k*), *w_b_*(*k*)), where *k* = *1*…*T*, *w_a_*(*k*) ∈ {*1*…*N* } and *w_b_*(*k*) ∈ {*1*…*M* }. This path remaps data points of *A* and *B*, by minimizing their distance following the condition that the first and last data points of the two time series are aligned. Other constraints such as monotonicity and step size are imposed on the function *w* to ensure reasonable wraps. The mean normalized distance (*mnd*) is then calculated using the following Equation:

(4) $${\text{mnd}}_{w}(A,B)=\sum_{k=1}^{T}\frac{d(w_{a}(k),w_{b}(k))c_{w}}{C_{w}}$$

where *c_w_* is a per-step weighting coefficient and *c_w_* is the corresponding normalization coefficient. Finally, to find the optimal path the minimum global dissimilarity is calculated using Dynamic Programming which breaks the entire set of solutions in sub-solutions thus reducing number of computations:

(5) $$\text{mgd}\;(A,B)=\underset{w}{\text{min}}\;{\text{mnd}}_{w}\;(A,B)$$

In our work, the *mgd* values of the first and second parts of the *MTS* and *Δt* time series were first extracted separately and then their absolute differences were used as similarity measures to quantify progressive reduction of tapping speed and tapping reaction time over the test trial, respectively. These two parameters are hence on denoted DTWMTS12 and DTWDT12.

The last parameter is designed to measure the overall trend of tapping reaction time over the test trial by calculating the mean correlation coefficient for jackknife (leave out one observation) samples between *Δt* and the corresponding timestamp sequences in milliseconds. The resulting parameter is hence on denoted MCDTT. Finally, in order to reduce the dimensions of these seven parameters and obtain a single score, PCA was applied. The first PC accounted for 35% of the variance in the data and was used to represent the A-FATIGUE.

#### 2.4.4. Calculation of Automated Arrhythmia Score (A-ARRHYTHMIA)

‘Arrhythmia’ in tapping is characterized by a serial irregularity in tapping performance, followed by an unpredictable behavior and abrupt changing patterns. In order to quantitatively measure tapping ‘arrhythmia’, the following parameters were calculated. The first two parameters were based on the application of the ApEn method. To quantify the presence of serial irregularity in tapping speed and vertical tap distance over the test trial, ApEn (with *m* = 2 and *r* = 0.2) was applied to *MTS* and raw y-coordinate time series, respectively. This resulted in two parameters, namely AEMTS and AEY.

In order to measure variation in distance between the two fields on the screen, the shimmer measure was calculated. Initially, a zero-crossing signal *ZC_1_*_…_*_n_* was constructed by finding the centre coordinate (*x_c_*, *y_c_*) between the two fields, followed by calculation of an Euclidean distance between centre coordinate and tap position (*x_t_*, *y_t_*) at each time interval *i*, using the following Equation:

(6) $$ZC_{i}=\{\begin{array}{cc} -\sqrt{{\left(x_{t_{i}}-x_{c}\right)}^{2}+{\left(y_{t_{i}}-y_{c}\right)}^{2}}, & if\;ZC_{i}<0\\ \sqrt{{\left(x_{t_{i}}-x_{c}\right)}^{2}+{\left(y_{t_{i}}-y_{c}\right)}^{2}}, & if\;ZC_{i}\geq 0 \end{array}$$

where *i* = *1*…*n* and *n* is the total number of time frames. The ZC signal was defined in order to measure variations in distance and time from one button to the other. Rhythmic tapping is associated with a relatively periodic ZC signal whereas arrhythmic tapping is associated with irregular patterns in time as well as distance, reflecting the patient's disability to keep the same timing and distance frequencies over the test trial. A normalized shimmer signal *S_i_* was computed by subtracting the absolute values of *ZC_i_* with the minimum value of the whole signal, followed by dividing the result with the absolute maximum value using the following Equation:

(7) $$S_{i}=\frac{\left|ZC_{i}\right|-\text{min}\left|ZC_{i}\right|}{\text{max}\left|ZC_{i}\right|}$$

The standard deviation of *S_i_* was calculated to measure the irregularity caused by distance variations during actual tapping, resulting in a new parameter denoted SDSHIM. Two more parameters used in computing ‘arrhythmia’ were based on the variation in jitter and shimmer. Jitter (*J_i_*) is a measure of irregularity computed using the time delays in the *ZC* signal as follows:

(8) $$J_{i}=\frac{\left|\Delta t_{i}\right|-\text{min}\left|\Delta t_{i}\right|}{\text{max}\left|\Delta t_{i}\right|}$$

where *Δt_i_* is the change in time between successive taps. Initially, variation in jitter and shimmer (VJS) was calculated to represent a tapping variation focusing on both the distance and time, using the following Equation:

(9) $${\text{VJS}}_{i}=\left|S_{i}-J_{i}\right|$$

The mean and standard deviation of *VJS* were calculated and used in subsequent analysis. These two parameters are hence on denoted MVJS and SDVJS.

A clinician, rating ‘arrhythmia’ using visualized graphs, would rate a sample as normal if he observes periodic patterns. On the other hand, he would rate a sample as extremely severe arrhythmic if he notices aperiodic patterns. The new parameter called the cross-correlation between the slopes (CCBS) quantitatively measures this aperiodicity by initially creating an artificial perfectly-periodic slope (PPS) signal using the *ZC* signal and then mapping it to the actual tapping signal. A distance series *dc_i_* signal was first calculated for taps *i* = *1*…*n* using:

(10) $${dc}_{i+1}=\{\begin{array}{cc} -{ZC}_{\text{avg}}, & d_{i}<0\\ {ZC}_{\text{avg}}, & d_{i}\geq 0 \end{array}$$

where *ZC_avg_* is the average distance between the taps in the *ZC* signal. Going further, the time variable was kept constant and taken as the average time delay *Δt_avg_* between the taps in the *ZC* signal and a time series signal was computed for taps *i* = *1* …*n* using:

(11) $$\Delta t_{i+1}=\Delta t_{\text{avg}}+\Delta t_{i}$$

The PPS signal was then constructed using both distance and time series signals as follows:

(12) $${\text{PPS}}_{i}=\frac{{dc}_{i+1}-{dc}_{i}}{\Delta t_{i+1}-\Delta t_{i}}$$

The original slope signal (OS) was then computed from the *ZC* signal by computing the slopes between the distance and time, using the following Equation:

(13) $${OS}_{i}=\frac{d_{i+1}-d_{i}}{t_{i+1}-t_{i}}$$

**Figure 3.**
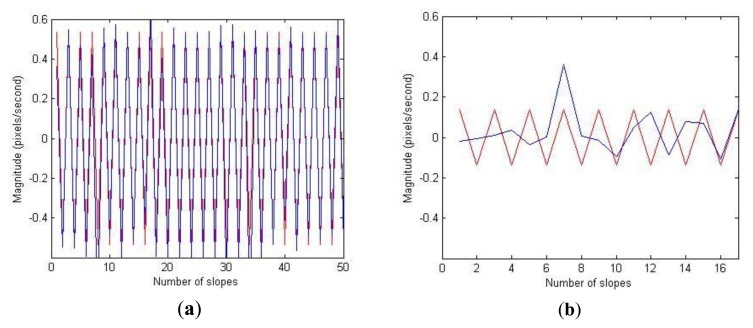
(**a**) The artificial perfectly-periodic slope (PPS) signal is superimposed on original slope (OS) signal for the case with V-GTS rated 0 (Normal). (**b**) The PPS is superimposed on OS for the case with V-GTS rated 4 (Extremely severe). The red line represents PPS and the blue line represents the OS.

Figure 3 shows superimposition of these two signals for two representative test occasions rated with GTS as 0 (normal) and 4 (extremely severe), respectively. The case rated as normal has relatively perfect overlap between the peak data points of the two signals whereas the case rated as extremely severe shows no overlap. In order to measure similarity between these two signals, initially a cross-correlation was computed and then the absolute mean value of the cross-correlation sequence was used as a measure to estimate rhythm in tapping.

The last parameter to quantitatively measure ‘arrhythmia’ was based on the cross-approximate entropy (Cross-ApEn) between PPS and OS. The Cross-ApEn quantifies the regularity of patterns in a pair of time series [25]. In contrast to ApEn, Cross-ApEn is applied to two signals and thus measures the dissimilarity between them. In addition, Cross-ApEn evaluates both spatial and temporal dissimilarities whereas the ApEn reflects only the temporal irregularity. Similarly as in the case of ApEn, the input parameters *m* and *r* were set to 2 and 0.2 (20% of the time series' standard deviation), respectively. The output of Cross-ApEn was used in the subsequent analysis and hence on denoted CABS. Finally, the PCA was applied to these seven parameters and the first PC accounting 35% of the variance in the data and was used to represent the A-ARRHYTHMIA.

#### 2.4.5. Calculation of Automated GTS Score (A-GTS)

In order to classify ATP based on the five GTS levels, a simple logistic regression model was used as a classifier to map the extracted quantitative parameters to the corresponding V-GTS scores. Initially, the PCA was applied to all the extracted parameters in order to reduce their dimensions, without much loss of variance in the data. An important step when applying PCA is to identify and retain the important components that account for a large proportion of the total variance. In this work, the appropriate number of “significant” components was decided by selecting a cumulative percentage of total variance for which it was desired that the selected PCs should account for more than 70% of the total variance in the original data. Applying this criterion resulted in retention of the first 5 PCs to be used as predictors in the subsequent regression analysis (Table 2). Dimensionality reduction with PCA helps in operating with a smaller number of variables in the subsequent analysis as well as to avoid the problem of multi-collinearity when using PCs as independent variables in the regression model. The output of the regression-based classifier was used to represent the A-GTS.

### 2.5. Data Analysis

Agreements between V-GTS and A-GTS were evaluated using the area under the receiver operating characteristics curve (AUC) and weighted Kappa statistics as major performance evaluation measures. A stratified 10-fold cross-validation (also known as rotation estimation) was applied to assess the generalization ability of the logistic regression classifier to future independent data sets. Spearman's rank correlation coefficients were used for assessing linear relationships between computed and visual scores. Reliability *i.e.*, internal consistency of the four tapping dimensions was assessed using Cronbach's *α* test. Sensitivity to treatment interventions and disease progression over time was assessed by evaluating changes in mean automated dimension scores of LCIG-naïve patients over time *i.e.*, at baseline and follow-up test periods, with linear mixed-effects models [26] using a restricted maximum likelihood estimation method with patient ID as a random effect and test period as a fixed effect of interest. The linear mixed-effects models were also used to (i) assess the ability to discriminate between healthy elderly subjects and the two patient groups, with subject ID as a random effect and group as a fixed effect of interest and (ii) assess differences in mean scores of the First PC relative to categories of the items #23–#25 of the UPDRS, with patient ID as random effect and category as a fixed effect of interest. Tukey *post-hoc* multiple comparison tests were performed to determine differences between subject groups. Inter-subject variability of the automated dimension scores was assessed using intra-class correlation coefficients.

## 3. Results

### 3.1. Correlations/Agreements to Visual/Clinical Scores

**Figure 4.**
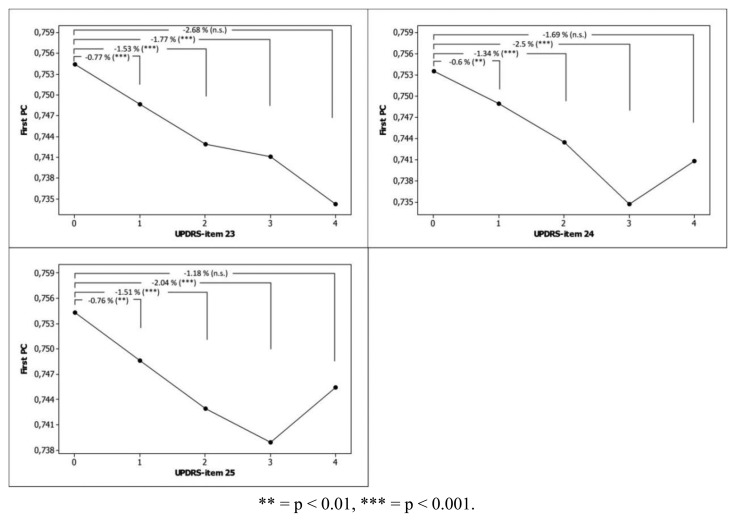
Mean scores of First PC for each category of items #23 (Finger Tapping), #24 (Hand Movements) and #25 (Rapid Alternating Movements of Hands) of the UPDRS scale, corrected for individual subject variation using linear-mixed effects models. Y-axis: a high score means good function. UPDRS items: 0 (Normal), 1 (Mild slowing and/or reduction in amplitude), 2 (Moderately impaired), 3 (Severely impaired) and 4 (Can barely perform the task). P-values and % changes are shown with respect to category 0. UPDRS item #23: 0 (n = 2323), 1 (n = 3649), 2 (n = 2793), 3 (n = 687), 4 (n = 25). UPDRS item #24: 0 (n = 2532), 1 (n = 3964), 2 (n = 2441), 3 (n = 491), 4 (n = 49). UPDRS item #25: 0 (n = 2201), 1 (n = 4085), 2 (n = 2511), 3 (n = 598), 4 (n = 82). Note that the category 4 of the UPDRS items had small number of observations that were assessed in very few patients.

The agreements between V-GTS and A-GTS were very good with a Kappa coefficient of 0.87 (p < 0.001) and weighted AUC value of 0.86 (Table 3). The best agreements were seen at extreme classes that is class 0 (normal, AUC = 0.93) and class 4 (extremely severe, AUC = 0.95). Correlations between computed and visual scores were strong (Table 4). The mean scores of the First PC for each category of items #23–#25 of the UPDRS scale are displayed in Figure 4.

### 3.2. Reliability

The internal consistency among the four automated dimensions was acceptable (Cronbach's *α* coefficient = 0.75), indicating that they measure the same underlying construct of the ATP.

### 3.3. Sensitivity to Change

**Figure 5.**
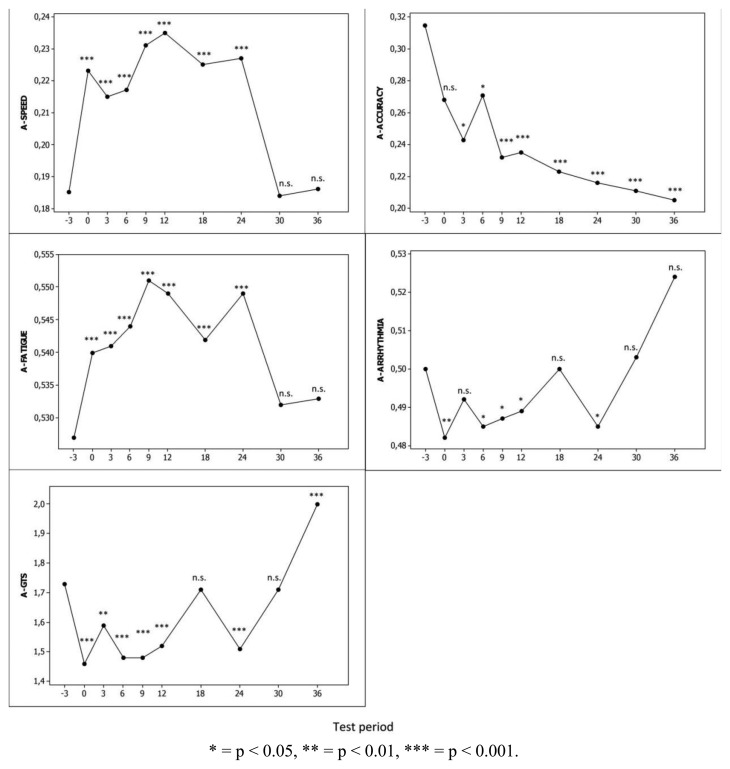
Trends of mean computed scores of LCIG-naïve patients over the 36-months study period, corrected for individual subject variation using linear mixed-effects models. Y-axis: a high score for A-SPEED, A-ACCURACY, A-FATIGUE and A-ARRHYTHMIA means good function whereas a high score for A-GTS means severe. P-values are shown with respect to baseline (−3) test period. Test period: −3, baseline (n = 507); 0 (n = 506); 3 (n = 468); 6 (n = 389); 9 (n = 417); 12 (n = 362); 18 (n = 296); 24 (n = 322); 30 (n = 227); 36 (n = 108).

Mean computed scores of the LCIG-naïve patients from baseline to the 36-month follow-up are shown in Figure 5. Mean A-SPEED, A-FATIGUE and A-GTS scores improved to the first test period on LCIG treatment and this improvement remained statistically significant until month 24 (p < 0.001). The mean scores of A-ARRHYTHMIA dimension deteriorated but only at different test periods. In contrast, mean A-ACCURACY scores deteriorated throughout the study period along with the natural disease progression.

### 3.4. Ability to Discriminate between Subject Groups

**Figure 6.**
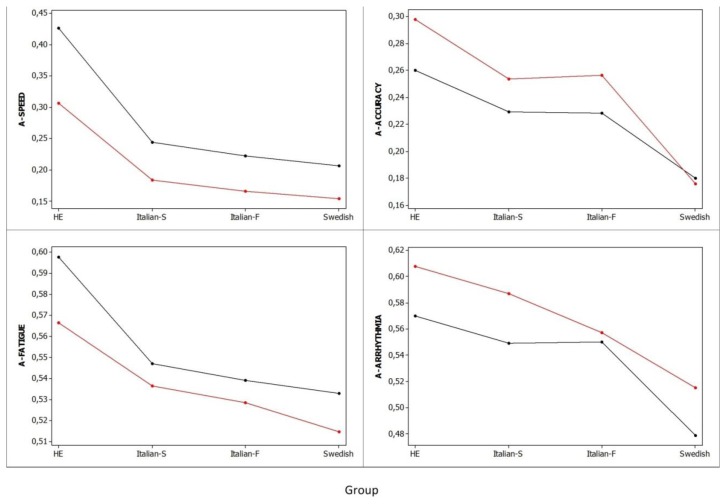
Mean scores of the four automated dimensions for each group, corrected for individual subject variation using mixed-effects models (black line: right hand; red line: left hand). Y-axis: a high score means good function. Group: healthy elderly, HE (n = 286); Italian S (n = 806); Italian F (n = 784); Swedish (n = 9,531).

Figure 6 summarizes the mean scores of the four automated dimensions for each subject group. Significant differences were found between HE and patient groups in all four dimensions. In general, HE had better tapping dimension scores than advanced patients with a 4,590% difference in A-SPEED, a 244% difference in A-ACCURACY, a 464% difference in A-FATIGUE, and 273% difference in A-ARRHYTHMIA. There were no significant differences in tapping dimensions between patient groups, except for better A-ACCURACY in early patients *i.e.*, Italian S and Italian F than in advanced Swedish patients with a difference of 96.9% and 95.1%, respectively (p < 0.05). The variability (the lower the variability, the closer to 1.0 the value of the intra-class correlation coefficient is) of dimensions in advanced Swedish patients was generally greater than that observed in HE (A-SPEED, 0.52 *vs.* 0.5; A-ACCURACY, 0.58 *vs.* 0.65; A-FATIGUE, 0.62 *vs.* 0.63; A-ARHYTHMIA, 0.59 *vs.* 0.66). The trends of mean scores of the automated dimensions for the two hands did not differ.

## 4. Discussion and Conclusion

In this study, we showed that quantitative and objective measures of ATP on a touch-pad test battery are valid measures of upper limb motor performance in PD. Majority of these measures had strong and significant correlations to visually assessed and clinical scores, suggesting that they contain important elements of symptom severity information in ATP. The regression-based classifier could classify the GTS of patients on a 0 (normal)–4 (extremely severe) scale comparatively well to the neurologist with a weighted Kappa coefficient of 0.87 and a weighted AUC of 0.86. The strongest correlations between computed and visual scores were seen when assessing GTS (0.91), ‘speed’ (0.89) and ‘accuracy’ (0.77). However, there were moderate and weak correlations when assessing ‘arrhythmia’ (0.57) and ‘fatigue’ (0.38), respectively. This possibly could be adjusted by adding more parameters measuring these two dimensions.

The main idea behind defining the four dimensions was to measure the severity of symptoms during tapping tasks as being represented in the items #23–#25 of the UPDRS scale [23]. Using these items, the marked severity in bradykinesia is rated in the beginning of the severity scale (“1 = Mild slowing and/or reduction in amplitude”) whereas the increased variability in time-dependent effects such as fatigue and arrhythmia is rated in higher categories *i.e.*, 2 (“Moderately impaired. Definite and early fatiguing. May have occasional arrests in movement) and 3 (“Severely impaired. Frequent hesitation in initiating movements or arrests in ongoing movement”). The proposed computer method combined the four dimensions in a data-driven approach where each dimension contributed to the assessment of the GTS score. When comparing mean computed scores across the 0–4 categories of the three UPDRS motor items, it was found that the mean scores of the First PC were significantly different.

The rationale behind including the Italian and HE datasets in the analysis was to have data from more early PD patients and healthy elderly, respectively along with the advanced PD patients from the Swedish study. Although having small sample sizes, adding these two datasets would assist in interpretation of the presented results. However, the employment of linear mixed-effects models allowed us to use all the data available, account dependencies within- and between-subjects, and model mean computed scores, with subject ID as random effects [27]. In addition, these methods are appropriate for analyzing repeated measures (longitudinal) data by accounting intra-subject correlation of measurements [26]. On average, HE had significantly better scores than PD patients in all four dimensions. The highest difference was seen at the A-SPEED dimension indicating that ‘speed’ measures are probably the most relevant ones when separating HE and early PD patients, which is expected because bradykinesia is a cardinal and an early symptom. Nevertheless when comparing early and advanced PD patients the only significant difference was observed at the A-ACCURACY dimension. In general, advanced Swedish patients had slightly better tapping results with the right hand than with the left hand. Sixty-two out 65 (95%) of them were right-handed. In a previous study [28], tapping scores (speed, calculated as number of taps per 20 s and accuracy, calculated as percentage of correct taps) were compared between patients with more affected right side (n = 36) and patients with more affected left side (n = 26). The results showed that the effect of handedness was more prominent than the effect of the side in which PD symptoms started *i.e.*, they had better tapping speed and accuracy scores during tapping test with the right hand than during tapping test with the left hand.

The PCA for the 24 parameters showed that the ATP could be explained by only 5 components (Table 2). Among the four dimensions, ‘speed’ had the highest number of measures which more contributed to the First PC compared to other dimensions, demonstrating that ‘speed’ is the key marker when assessing ATP of patients. The unsupervised approach of defining the First PC showed to correlate well with visual measures of ATP; V-GTS (0.88), V-SPEED (0.91), V-ACCURACY (0.55), V-FATIGUE (0.58) and V-ARRHYTHMIA (0.55). The within-subject variability on repeated measures indicated that advanced PD patients had a higher variability in their A-ACCURACY and A-FATIGUE scores compared to HE subjects. Possible reasons for this greater variability include: greater heterogeneity of symptom profiles among and within advanced/fluctuating patients, improvement or deterioration of symptoms as a result of treatment changes and expected natural progression of symptoms during a relatively long time that is 36 months.

The method also showed to be sensitive to treatment interventions. The significant improvements in mean A-SPEED, A-ACCURACY and A-GTS scores indicated that the method was able to measure motor symptom improvements with LCIG that were sustained over at least 24 months. These changes were also documented with the clinical rating scales [29]. In contrast to other dimensions, A-ARRHYTHMIA and A-ACCURACY did not improve reflecting the expected natural progression of PD over time. These results are in line with the results from our previous research which showed that in contrast to tapping speed, the tapping accuracy progressively deteriorated during the 36-months study period of advanced patients [30]. It was also found that there was an earlier deterioration of tapping speed compared to tapping accuracy indicating that worsening of tapping accuracy could become a marker for considering advanced PD treatments [28].

A limitation of the present study is that the clinical evaluation of ATP is done by visual inspection of graphs and not by live/video observations of the patient's performance. However, even these kinds of observations may be biased as a result of the within- and between-clinician variability in ratings. In the study performed by Heldman *et al.* [31], it was shown that clinicians differentially weighed movement components of speed, amplitude and rhythm during video recorded tasks on items #23–#25 of the UPDRS scale. This illustrates that the whole-body video sequence may give a clinical impression of symptoms, e.g. axial hypokinesia, that are not necessarily reflected in upper limb motor performance. In this study, the rationale for visualizing graphs of ATP to clinicians was to derive a target measure to be used in correlation/agreement analysis against computed measures in order to develop the method. The next step in our research would be to gather video recordings of patient's performance during tapping tests along with telemetry measurements of the test battery. The video recorded test occasions then will be clinically evaluated leading to assessment of the feasibility of visualizing graphs to clinicians as well as to validate the computer-based approach. However, it is important that the computed scores of ATP correlated well to visually assessed scores, were significantly different across UPDRS motor ratings of upper limb motor performance, had good internal consistency, had good ability to discriminate between healthy elderly subjects and patients in different disease stages, had good sensitivity to treatment interventions and were able to reflect the natural PD progression over time.

PD is a multidimensional and complex disorder affecting both motor and non-motor symptoms. The overall well-being and the quality of life of PD patients were shown to be highly influenced by non-motor symptoms and weakly by motor symptoms [32]. Therefore, in order to obtain a reliable assessment of the degree of the patient's disability, it is essential to account for both these two types of information. The main aim of the telemetry test battery is to combine subjective measures, which target patients' perception towards their symptoms, and objective measures of fine motor function (tapping and spiral drawing [33]), which target the actual physiological functioning, into composite scores for representing different symptom severities as well as the global health condition and disability of the patient over week-long test periods [34]. The derived scores from this study provided means for objective characterization of kinematics and dynamic performance during tapping tests in the test battery. The compliance with the telemetry test battery was previously assessed [20,21] as the number of completed test occasions per test period. The results indicated that patients were generally compliant with using this technology with median compliance of 93%.

In summary, the method we developed for the alternate tapping test is appropriate to quantitatively and objectively assess the severity of ATP of PD patients. The clinimetric properties, *i.e.*, correlations to visual ratings, reliability and sensitivity to treatment changes and to natural disease progression over time, of the method indicate that it can be included in tools for repeated and remote monitoring of the said fine motor performance.

## Figures and Tables

**Table 1.** Characteristics of PD patients and of healthy elderly participants, presented as median ± interquartile range.

	**Swedish Study**	**Italian Study** **(F Group)**	**Italian Study** **(S Group)**	**HE** **(Healthy Elderly)**
Patients (n, gender)	65 (43 m; 22 f)	15 (13 m; 2 f)	15 (13 m; 2 f)	10 (5 m; 5 f)
Age (years)	65 ± 11	65 ± 6	65 ± 6	61 ± 7
Years on levodopa	13 ± 7	7 ± 8.5	5.5 ± 6	NA (not applicable)
Hoehn and Yahr stage at present	2.5 ± 1*	2 ± 0 **	2 ± 0.5	NA (not applicable)
Total UPDRS	49 ± 20.5*	33.5 ± 11.8 **	26 ± 16.5	NA (not applicable)

* Assessments performed in afternoons;

** Assessments performed in on-state.

**Table 2.** Percentage total variance accounted for by first 5 PCs and contributions of the 24 parameters in each one of them. Details of the parameters are discussed in the text.

**Parameter**	**Dimension**	**First** **PC (%)**	**Second** **PC (%)**	**Third** **PC (%)**	**Fourth** **PC (%)**	**Fifth** **PC (%)**
TNT	‘speed’	6.8	0.2	1.9	4.3	2
MTS	‘speed’	6.9	3.5	0.3	1	0.7
MTSLR	‘speed’	6.6	3.5	1.9	2	4.1
CVTSLR	‘speed’	2.7	5.9	4.9	1.5	14
MTSRL	‘speed’	6.7	3.1	1.7	0.3	3.7
CVTSRL	‘speed’	2.5	7.7	2.2	7.4	3
MDCF	‘accuracy’	3.3	6.2	6.8	8	1.8
CVDCF	‘accuracy’	1.4	5.8	8.8	9.7	0.2
ODT	‘accuracy’	5.5	5.5	0.5	2.3	0.2
OTP	‘accuracy’	6.1	4.5	2	1.1	1.2
MTSPC	‘fatigue’	6.9	1.5	0.7	3.2	2.2
DDT12	‘fatigue’	4	6.1	9.5	1.5	6.2
DMTSPC12	‘fatigue’	2.8	8.1	1.4	5.3	0.6
DAEDT12	‘fatigue’	1.6	0.8	8	7	12.4
DTWMTS12	‘fatigue’	4.4	7.2	0.3	7.9	1.6
DTWDT12	‘fatigue’	3	5.5	10.6	4.9	10.1
MCDTT	‘fatigue’	0.4	0.3	12.6	4.7	4.6
AEMTS	‘arrhythmia’	4.9	1.2	0.3	10.1	3.8
AEY	‘arrhythmia’	5.3	3.3	2.8	4.7	3.6
SDSHIM	‘arrhythmia’	4.6	3.2	2.3	1.3	6.6
MJVIS	‘arrhythmia’	1.6	0.3	11.4	2.2	2.4
SDVJS	‘arrhythmia’	4.2	4.9	4.3	2.1	7.3
CCBS	‘arrhythmia’	6	4.7	1.6	2.4	0.7
CABS	‘arrhythmia’	1.8	6.9	3.3	5	6.8
Total variance	NA (not applicable)	39	16	7	6	4

**Table 3.** Assessments of GTS for the computer method and human rater. The computed scores are derived after applying 10-fold cross validation on the logistic regression classifier.

		**Computer**					

		**0**	**1**	**2**	**3**	**4**	**Total/Weighted**
**Human Rater**	**0**	14	6	0	0	0	20
	**1**	7	10	4	0	0	21
	**2**	2	5	7	6	0	20
	**3**	0	0	8	8	3	19
	**4**	0	0	0	3	14	17
	**Total**	23	21	19	17	17	97
	**AUC**	0.93	0.82	0.74	0.85	0.95	0.86

**Table 4.** Absolute Spearman rank correlations between computed and visual scores.

	**A-GTS**	**A-SPEED**	**A-ACCURACY**	**A-FATIGUE**	**A-ARRHYTHMIA**	**First PC**
A-GTS	1	0.69	0.58	0.56	0.72	0.92
V-GTS	0.91	0.89	0.55	0.62	0.65	0.88
V-SPEED	0.89	0.89	0.65	0.81	0.80	0.91
V-ACCURACY	0.59	0.39	0.77	0.32	0.54	0.55
V-FATIGUE	0.57	0.49	0.53	0.38	0.54	0.58
V-ARRHYTHMIA	0.63	0.34	0.64	0.54	0.57	0.55
